# Heterogeneity in Colorectal Cancer: A Challenge for Personalized Medicine?

**DOI:** 10.3390/ijms19123733

**Published:** 2018-11-23

**Authors:** Chiara Molinari, Giorgia Marisi, Alessandro Passardi, Laura Matteucci, Giulia De Maio, Paola Ulivi

**Affiliations:** 1 Biosciences Laboratory, Istituto Scientifico Romagnolo per lo Studio e la Cura dei Tumori (IRST) IRCCS, 47014 Meldola, Italy; chiara.molinari@irst.emr.it (C.M.); giorgia.marisi@irst.emr.it (G.M.); giulia.demaio86@gmail.com (G.D.M.); 2 Department of Medical Oncology, Istituto Scientifico Romagnolo per lo Studio e la Cura dei Tumori (IRST) IRCCS, 47014 Meldola, Italy; alessandro.passardi@irst.emr.it (A.P.); laura.matteucci@irst.emr.it (L.M.)

**Keywords:** heterogeneity, colorectal cancer, tumor tissue, metastasis, cfDNA, liquid biopsy, response to therapy

## Abstract

High inter-patient variability and high spatial heterogeneity are features of colorectal cancer (CRC). This may influence the molecular characterization of tumor tissue, now mandatory for patients with metastatic CRC who are candidates for treatment with an anti-EGFR mAb, as false-negative results can occur, leading to non optimal therapy. Moreover, temporal molecular heterogeneity during treatment is known to influence the response to therapy and prognosis. We present a literature overview of advances made in characterizing molecular heterogeneity in CRC, underlining that the analysis of liquid biopsy could represent an efficient non-invasive tool to overcome the problem. We believe that understanding CRC heterogeneity is fundamental for a more accurate diagnosis, for selecting the best targets to ensure prolonged antitumor response, and for monitoring minimal residual disease and the onset of resistance to therapy, all essential components of successful personalized treatment.

## 1. Introduction

Colorectal cancer (CRC) is the third most commonly diagnosed cancer in males and the second in females, with 1.7 million new cases and almost 832,000 deaths worldwide in 2016 [1]. It has been estimated that more than 20% of patients present with metastatic CRC (mCRC), and around 50% of patients with localized CRC will develop metastases [2]. In Europe the overall survival (OS) rate for mCRC is estimated at 12%, with a median OS of 30 months [3]. A mortality rate of 15.8/100,000 for men and 9.2/100,000 for women has been predicted for 2018 [4]. 

mCRC may be resectable or non resectable. Up to 30% of patients can be cured if metastases to the liver are completely removed. Chemotherapy (CT) may be recommended before surgery if metastatic disease is confined to the liver or if there is a limited metastatic disease in the lung [5]. If surgical removal of the liver metastases is successful, additional chemotherapy is often recommended. However, in the majority of cases, surgery is not possible and CT is recommended to reduce symptoms and prolong survival. Fluoropyrimidine-based chemotherapy regimens in combination with antiangiogenic agents or monoclonal antibodies directed against epidermal growth factor receptor (anti-EGFR mAb) are approved worldwide for first-line treatment of the disease. The determination of *KRAS*, *NRAS*, and *BRAF* mutational status is essential when metastatic disease is diagnosed to optimize the choice and sequencing of therapy.

In this era of precision medicine, it must be taken into account that CRC is characterized by high inter-patient heterogeneity and high intra-tumor heterogeneity (ITH), given the remarkable genomic instability (microsatellite and genomic instability) and field cancerization of the disease [6,7]. Moreover, there is evidence that exogenous factors such as lifestyle, diet, nutrition, environment, and microbiome contribute to pathogenesis, also influencing non neoplastic cells, including immune cells, and leading to further heterogeneity [8,9]. In this context, the importance of investigating the interactive effects of tumor molecular changes and the totality of exposures (called exposome) that differ from individual to individual has now been acknowledged. This has become a new research field known as molecular pathological epidemiology (MPE), whose aim is to provide novel insights into interactions among the environment, tumor, and host to better understand how endogenous and exogenous factors modify tumor phenotypes [10,11]. 

Many of the genome-scale studies on heterogeneity are limited to the analysis of primary cancers and normal paired tissues, and further research into metastatic disease is needed [12,13]. Indeed, data on the concordance between the genes analyzed in the primary tumor and in paired metastases are increasing thanks to -omics technology, but are sometimes conflicting [14]. The need to better characterize the molecular features of CRC heterogeneity has also been acknowledged given the varied response of patients with similar phenotypes to therapy [15]. 

In the present review we focus on tumor heterogeneity in CRC (both primary and metastatic) and describe its impact on response to treatment and prognosis. We also discuss its evolution during treatment, paying particular attention to the role of liquid biopsy in tumor heterogeneity characterization and monitoring. 

## 2. Inter-Patient Heterogeneity

The majority of sporadic CRCs (almost 85%) arise from adenomas according to the model proposed by Fearon and Vogelstein in 1990 [16]. They are characterized by chromosomal instability (CIN), a negative CpG island methylator phenotype (CIMP), microsatellite stability (MSS), and a *KRAS*-mutated and *BRAF* wild type (wt) gene. Conversely, about 15% of CRCs originate from serrated adenomas following a pathway characterized by CIMP-high, microsatellite instability (MSI) and *BRAF* mutation [17]. A subset of these tumors possess markedly elevated mutation rates, being characterized by dysfunction of mismatch repair (dMMR) genes leading to high MSI (MSI-H). MSI-H tumors make up a minority of CRCs, with a favorable prognosis in early-stage disease and a lower frequency in more advanced stages [18]. Interestingly, some authors have hypothesized that even mCRC patients with *KRAS* mutations may be a heterogeneous population, reporting that only patients with *KRAS* G13D mutation rather than *KRAS* G12R mutation show a survival benefit from treatment with targeted therapy combined with chemotherapy [19]. Molecular and phenotypic differences also exist in relation to tumor localization. The different origins of left- and right-sided tumors lead to different gene expression and mutation profiles, ultimately influencing prognosis [20]. In particular, right-sided tumors show a higher frequency of *BRAF* mutations and MSI, and tend to develop in patients with a genetic predisposition to CRC (e.g., Lynch syndrome). Conversely, left-sided tumors are characterized by CIN and a gene expression profile involving the activation of the EGFR pathway [20,21,22]. In addition, a significantly higher number of mutations are present in right- compared to left-sided tumors (102 vs. 66, *p* = 0.004) [23]. Ulivi et al. also reported a different distribution of angiogenic and inflammatory markers in relation to tumor localization. In particular, higher expression levels of cyclooxygenase-2 (COX2), ephrin type-B receptor 4 (EPHB4), and endothelial nitric oxide synthase (eNOS) were observed in right-sided with respect to left-sided tumors, whereas baseline inflammatory indexes such as neutrophil-to-lymphocyte ratio, platelet-lymphocyte rate, and systemic immune-inflammation index were higher in the latter [24]. Finally, the microbial richness and composition that is believed to play an important part in the formation of CRC vary on the basis of the primary tumor location [25].

All of these differences lead to a heterogeneous sensitivity to treatment and to a varied prognosis [20,26,27,28].

CRC is known to be characterized by a high number of mutations, but only 20% are drivers of the disease, and even fewer are common to more than one tumor. Moreover, as with other tumors, only a weak correlation exists between somatic mutations and phenotype expression [12]. The concept of consensus molecular subtypes (CMS) was proposed by Guinney et al. to classify CRCs into four subtypes [12,22,29]. CMS1 is enriched in MSI tumors with an immune activation and is known as the “MSI immune” subtype. CMS2 tumors display epithelial characteristics and upregulation of WNT and MYC downstream targets, while CMS3, the metabolic subtype, includes *KRAS*-mutated tumors with the activation of metabolic pathways. Finally, CMS4, known as the mesenchymal subtype, includes tumors with mesenchymal features showing a high stromal content and activation of tumor growth factor beta (TGF-β) and vascular endothelial growth factor receptor (VEGFR) pathways. In addition to all of the obvious biological differences, there are also clear clinical distinctions between the CRC subtypes, CMS4 tumors having a poor prognosis and CMS1 a relatively good one [15]. 

It appears that increased genomic instability as a result of an abnormal tumor microenvironment could further contribute to tumor cell diversification. In fact, a higher spatial and temporal variability of tumor microenvironment components, such as stromal, inflammatory, and microbial cells, may translate into a higher phenotypic heterogeneity of tumor cells [30]. This is in line with findings recently reported by Bramsen et al. who demonstrated that microbiome deeply influences tumor archetypes and is associated in particular with a new metabiome-dependent CIN subtype [8].

## 3. Spatial Heterogeneity in Colorectal Cancer 

In addition to inter-patient heterogeneity, three types of spatial heterogeneity are important in tumorigenesis. First, ITH may refer to differences in cells within the same primary tumor. Secondly, ITH may be at the basis of the inter-metastatic heterogeneity that consists of differences in various metastatic lesions in the same patient. Finally, distinct mutations can also co-exist within the cells of a single metastatic lesion [7]. This spatial heterogeneity is related to the presence of clones that are genetically distinct as a consequence of evolutionary processes, and also to the co-existence of fully-differentiated cancer cells and immature cancer stem cells within the same cancer [31]. As demonstrated by Kreso et al. in CRC, there may also be functional heterogeneity between tumor cells with a uniform genetic lineage, with cells differently capable of growing and responding to chemotherapy [32]. In addition, different immune cell distribution and action, influenced by local tumor cells, stroma, and microenvironmental factors, such as microbiota, contributes to spatial heterogeneity in CRC [9].

Five different models of CRC molecular evolution are described by Amaro et al. each model highlighting a different scenario about the possibility of predicting distant metastases by analyzing the primary tumor [31]. Among them, the “big bang” model proposed by Sottoriva et al. appears to fit in best with the mutation patterns observed in CRC. In Sottoriva’s study, more than 300 individual glands from 15 CRCs were profiled by massive genomic characterization, revealing that ITH derives from early passenger alterations rather than from clonal selection. The different clones generated by genomic instability are indicative of a highly heterogeneous primary cancer and are capable of stabilizing and moving through a linear evolution towards metastatization [33]. Thus, according to this model, the characterization of the bulk of the primary tumor may be sufficient for prognosis.

ITH leads to an underestimation of the mutational landscape portrayed by single needle biopsy, and consequently affects treatment accuracy. Thus, spatial heterogeneity is at the basis of resistance to treatment, especially when selective pressure has been exerted, for example, by drugs in late-stage cancers [34]. This is not only due to the wide-ranging mutagenic effect of chemotherapy, but also to the so-called “competitive release” induced by chemotherapy that allows treatment-resistant subclones to repopulate and drive the relapsed tumor [35,36]. 

Furthermore, some studies have suggested that heterogeneity, measured by the existence of multiple subclonal alterations within the same tumor, may be associated with poor outcomes in many cancers [37]. With regard to CRC patients, Sveen at al. demonstrated that intra-patient and inter-metastatic heterogeneity were strong prognostic determinants. Patients with a low level of heterogeneity had a three-year progression-free (PFS) and OS rate of 23% and 66%, respectively, compared to 5% and 18% for those with high heterogeneity (hazard ratio (HR) = 0.4, 95% confidence interval (95% CI) 0.2–0.8, *p* = 0.01; and HR = 0.3, 95% CI 0.1–0.7, *p* = 0.007; respectively) [38].

The lack of accurate standard methods to assess ITH is currently limiting our ability to explore its clinical implications [39]. As mentioned by Wang et al. in a recent review, single-cell sequencing and computational deconvolution of bulk-tumor gene expression profiles could represent important strategies for analyzing the link between inter- and intra-tumor heterogeneity, even though the accurate characterization of the tumor cell subpopulations remains a challenge. It has also been shown that multi-omic data integration at genetic, epigenetic, transcriptomic, and proteomic levels is needed to further understand the heterogeneity of primary and metastatic lesions [40].

Whole-exome sequencing or high-coverage multigene panels carried out on multiple biopsy sites are currently the standard procedure for assessing spatial heterogeneity. However, ultra-deep sequencing may be required for an accurate assessment of ITH. Digital PCR also has a high sensitivity, but is costly and technically difficult to use in these kinds of studies [41]. Apart from -omic studies aimed at further characterizing CRC, more targeted approaches could be used to improve patient management. Recently, NGS targeted panels were proposed for use in clinical practice as they are less expensive and time-consuming [42,43]. However, their usefulness is still open to discussion, and the genetic alterations limited to the spectrum of *RAS* and *BRAF* mutations are still the most widely analyzed markers. 

### 3.1. Molecular Differences within the Primary Tumor

Considering the ITH found in CRC, contradictory results published on the prognostic and predictive significance of the most common genetic alterations in these tumors may be a result of the analysis of only one small tumor area. In their study in the late 1990s, Baisse et al. demonstrated that 67% of the advanced CRCs analyzed had ITH when gene alterations and loss of heterozygosity (LOH) were examined in 15–20 areas within the tumor, indicating the importance of tumor sampling [44]. Later, numerous other studies revealed that, despite the frequently high degree of concordance between alterations in different tumor areas [45], analysis of a single tumor block led to the incorrect assignment of *KRAS* and *BRAF* status in 10–30% of cases [46,47,48]. Interestingly, as highlighted by Buttner et al. morphological heterogeneity detected by conventional histology is important in the molecular assessment of CRC, as it may be an indicator of genetic ITH. The authors suggested that all morphologically different components identified within a tumor must be included in the analysis in order to detect alterations that might be involved in primary drug resistance [49]. This is in line with results reported by Reggiani-Bonetti et al. who showed that biomolecular ITH in CRC may depend on the grade of differentiation of the tumor. These authors found that poorly differentiated clusters (PDC), which are the most likely to give rise to metastatic disease, have a different mutational status that overlaps more with nodal metastases than with the tumor, thus demonstrating that peculiar histologic features may indicate ITH [50].

In the majority of studies, a specific tissue map was not considered when tissue was formalin-fixed and paraffin-embedded, and so the heterogeneity between specific locations within the tumor could not be analyzed. There are still no guidelines available to assess the adequacy of tissue sampling in representing the molecular features of the entire tumor. This represents a particular challenge when preoperative treatment is considered, since the choice is often based on the status of small biopsies taken from the surface of the tumor and sometimes leads to inappropriate decisions. For this reason, it is recommended that at least two biopsies be taken from different parts of the tumor to take into account ITH and avoid mis-sampling [48].

In addition to tissue sampling, the sensitivity of the technique used to evaluate heterogeneity undoubtedly influences results. Laurent-Puig et al. using highly sensitive picodroplet digital PCR, suggested that in patients with mCRC showing *KRAS*-mutated subclones, ≤1% benefitted from anti-EGFR therapies, while others did not [51]. 

Suzuki et al. recently reported that a variable pattern of ITH was observed using deep-targeted NGS followed by ultra-deep amplicon sequencing in different CRC patients [41]. In their analysis of 24 specimens from 4 CRC patients, the authors found that the different tumor sectors not only shared mutations in driver genes including *APC*, *KRAS*, and *TP53*, but also many other mutations. However, numerous mutations were present at the subclonal level around the primary tumor and were only revealed by an ultra-high-depth sequencing approach. Among patients, more extensive ITH was observed in MSI tumors, probably depending on the higher mutational rate [41]. Li et al. analyzed 747 CRC samples by NGS to look for mutations in 22 cancer-related genes and found that intratumor mutational heterogeneity, estimated by mutant allele frequency and tumor cellularity, most often occurred in *PIK3CA* mutant tumors [52].

ITH also affects mitochondrial DNA (mtDNA), known to play a critical role in the initiation, metastatization, and metabolic changes of cancer cells. In fact, different mtDNA mutations were detected in multiple regions of 13 CRCs, in particular in tissue samples from the peripheral part of the tumor [53].

A different degree of heterogeneity has been observed in tumors at various stages of the disease. Losi et al. reported higher ITH in early CRC with respect to advanced stages in terms of *TP53* and *KRAS* point mutations (70% vs. 20% and 60% vs. 20%, respectively), with an almost complete absence of heterogeneity in distant metastases. However, this was not true for 5q and 18q allelic losses, suggesting that a high level of CIN is not compensated by clonal selection [54]. The reduction in ITH for point mutations and the relative stability of ITH in terms of copy number alterations (CNAs) observed during CRC progression observed by Losi et al. seem to be in line with the previously mentioned “big bang” model for CRC evolution proposed later by Sottoriva [33].

Yaeger et al. recently described very few genomic differences in various stages of CRC, with only *FBXW7* and *TP53* alterations significantly enriched in early stage tumors and metastatic disease, respectively. Furthermore, a high concordance between genomic alterations was demonstrated when multiple samples from the same patient were sequenced [55].

### 3.2. Molecular Differences between the Primary Tumor and Metastases

The liver is the most frequent site of distant spread of CRC because much of the intestinal mesenteric drainage enters the hepatic portal venous system. As a result, liver metastases are a major cause of death from CRC. Around 20% of patients present with synchronous liver metastases [56], while another 40% develop metachronous liver metastases after resection of the primary tumor [57]. The second most common metastatic site is the lung [58,59], followed by the peritoneum (around 10% of patients) [60,61]. Ovarian metastases occur in 5%–10% of all women with mCRC [62], while brain metastases are relatively rare, with a reported incidence of 1%–2% [63]. CRC originating in the left colon and rectum has a higher incidence of liver and lung metastases than that of right-sided cancer, which shows a higher rate of peritoneal metastases and other-site metastases [55]. Moreover, patients with liver metastases from right-sided CRC have a significantly worse OS than those with liver-metastasized left-sided colon cancer [64].

Several studies have explored the mutational concordance between the major pathway genes, including *RAS*, *BRAF*, and *PIK3CA*, of CRC tissue lesions through paired analysis of surgical specimens of primary and metastatic lesions, generating contradictory results. Concordance of mutational status was defined as either the absence or presence of the mutation in both the primary tumor and the matched metastases. The majority of studies mainly examined liver metastases, revealing that mutation profiles were highly concordant (>90%) between primary and metastatic lesions [13,23,38,65,66,67,68,69], regardless of the temporal nature of the metastases (synchronous or metachronous) [70,71,72]. However, some authors have suggested that synchronous primary CRC lesions have a high frequency of heterogeneity in *KRAS* mutations [45]. In their study of spatio-temporal changes in CRC by targeted NGS, Kovaleva et al. confirmed the conservation of *RAS* mutation status in primary CRC and paired metastases, albeit with a different allele frequency. However, multiple metastatic lesions of the lung resected at the same time may differ in their mutation profile, whereas this is not true for liver metastases. Interestingly, the authors revealed that a number of de novo mutations occurred in late disease stages of metachronous CRC lung metastases, several of which may be targetable by specific therapies [73]. Conversely, Lee et al. reported that about half of the liver lesions analyzed from mCRC showed genetic heterogeneity with respect to their corresponding primary CRC, indicating the need to evaluate metastatic lesions separately to optimize therapy rather than using primary tumor data [74]. Similarly, Vermaat et al. observed a substantial loss or gain of gene variants from primary tumors with respect to their metastases. This indicates that genetic analysis of metastases may have greater predictive power when patients are selected for specific treatment modalities, thus allowing for further refinement of treatment algorithms [75]. Other studies demonstrated that CNAs differed in primary tumor and metastases, but were rarely observed in CRC-specific genes [23].

With regard to peritoneal [72], brain [76], and ovarian metastases [77], several authors observed a high concordance rate (>95%) for driver genes between primary tumors and these metastases, whereas Tortola et al. reported a discordance between *KRAS* mutations in bone-marrow micro metastases and the primary CRC [78]. Interestingly, Watanabe et al. found that all the metastatic lesions from 25 patients with multiple liver metastases and 11 patients with multiple lung metastases showed identical *KRAS* mutations, highlighting the importance of *KRAS* status in the primary tumor [79]. However, despite the overall concordance rate of 93.5% between paired tumors and metastases regardless of their localization, Kim et al. observed that nearly all samples sequenced by NGS had at least one discordant mutation, with *APC* and *TP53* among the seven genes with two or more discordant alterations [13]. Beije et al. analyzed a panel of 21 target genes by NGS, reporting a concordance for all variants between the primary tumor and metastases of around 72% [80]. Mogensen et al. found that the number of shared mutations between primary CRC and synchronous liver metastases varied from 50% to 96% when whole-exome sequencing was performed, depending on the gene considered. No discordance was observed for *RAS* or *BRAF* mutations, while some private mutations in well-known driver genes, such as *PIK3CA* and *SMAD4*, were observed [23].

It would appear that the localization of metastases may, in itself, be an indicator of the risk of discordance. In their subanalysis of the concordance of *KRAS* between primary tumors and hepatic versus extrahepatic metastases, Baas et al. observed a concordance rate of 95% for hepatic metastases and of 86% for extrahepatic lesions (*p* = 0.01). In particular, discordance with the primary tumor occurred more frequently in lymph node metastases (95% vs 84%; *p* = 0.01), and concordance was lower in patients with primary *KRAS*-mutated tumors (95% vs. 86%; *p* =0.01) [66]. In agreement with these findings, Mao et al. reported that lymph node metastases showed a lower concordance rate of *KRAS* mutation status [14]. Similar results were obtained by Fujiyoshi et al. who also underlined the importance of considering the cellularity and degree of necrosis of samples, especially when analyzing lymph nodes [72].

Finally, in an analysis of mitochondrial DNA alterations, a higher discordance was observed between lymph node metastases and primary tumor with respect to distant metastases and primary tumor [81]. Overall, the higher discordance observed for lymph nodes lesions is in line with data recently published by Naxerova et al. who showed that 2 distinct patterns of metastatic dissemination exist in CRC. The authors found that in about 65% of mCRC patients, lymphatic and distant metastases were generated from independent subclones in the primary tumor, whereas only about one third of cases shared a common subclonal origin [82]. They thus hypothesized that, in the case of the former, cancers contain multiple genetically distinct metastasis ancestors. In particular, lymph node colonization may occur more frequently and earlier when, according to the big bang model, ITH may be higher [33,82]. Otherwise, liver metastases may arise in later stages of disease from more stable clones and therefore appear to have more mutations, but also to be more homogeneous [82]. Given that lung metastases are more frequently seeded through lymph nodes, the discordance of *KRAS* status observed for lung by Kim et al. [83] may be supported by this model. Mutated *KRAS* tumors developed lung metastases more frequently as the initial metastatic site, whereas the liver and distant lymph nodes tended to be the first sites of metastasis in wt *KRAS* tumors. However, other metastatic sites (e.g., peritoneum) would seem to be affected by *PIK3CA* mutations rather than by *KRAS* status [83,84].

## 4. Liquid Biopsy to Overcome Tumor Tissue Heterogeneity

**Figure 1:**
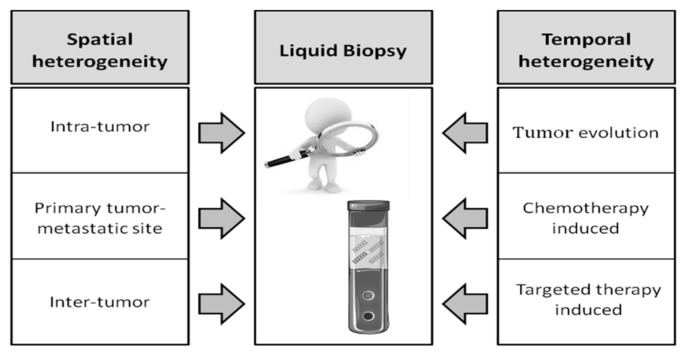
Overview of the main types of heterogeneity in colorectal cancer (CRC), and the central role of liquid biopsy.

Different tumor components (circulating tumor cells (CTCs), circulating tumor DNA (ctDNA), exosomes, microRNAs) isolated from body fluids through “liquid biopsy” can provide additional useful information for diagnostic, prognostic, and predictive purposes. The study of liquid biopsy has acquired a prominent place in cancer research, and one of the features that renders it especially promising is its capacity to overcome the problem of tumor heterogeneity (Figure 1). Assuming that tumor tissue is heterogeneous, a tissue biopsy performed in a particular part of the lesion may not be representative of the genetic landscape of the entire tumor. Liquid biopsy on the other hand is more widely representative of the entire lesion and enables tumor evolution to be followed in real time. In a recent review, Hench et al. discussed the advantages and limitations of CTC and ctDNA evaluation in breast, lung, and CRC, envisioning a potential role of liquid biopsy for second-line diagnosis and tumor surveillance, even though its analytical and clinical validity has yet to be definitively demonstrated [85].

ITH, multiple metastatic clones that disseminate and remain dormant for years, and methodological pitfalls may be responsible for the discordance often observed in *KRAS* status between CTCs and CRC tissue. Fabbri et al. reported a concordance of only 50% in their case series of 40 CRC patients [86]. Kondo et al. analyzed primary tumor tissue and CTCs in 11 patients, observing a concordance of around 64% in *KRAS* mutational status [87], while other studies obtained a concordance of 71–77% [88,89]. Of note, even *KRAS* status in CTCs from the same patient proved to be heterogeneous [90]. 

Given that cell-free (cfDNA) and ctDNA appear to have a strong prognostic value in mCRC and are directly related to disease burden, they might be useful to monitor disease from early stages through disease evolution under treatment. Interestingly, Bettegowda et al. observed that ctDNA was always detected even when no CTCs were found, whereas there were no instances when CTCs were present but ctDNA was absent [91].

Finally, the analysis of exosomal DNA or RNA to detect tumor-specific genetic mutations in CRC is a potentially promising area of research. Given its concentration and abundant content, exosomes could help to increase the sensitivity of liquid biopsy, despite the presence of some methodological limitations [92]. A high consistency rate (73.7% sensitivity and 100% specificity) has been seen for *KRAS* mutations detected using exosomes from serum [93]. Moreover, as it has been found that mutant *KRAS* rather than wt *KRAS* DNA is loaded preferably into exosomes, the analysis of these vesicles could likely facilitate patient stratification and thus improve clinical management [94].

### 4.1. Comparison between cfDNA and Tumor Tissue 

Several studies have been performed to compare gene mutations in cfDNA and tumor tissue (Table 1). The majority of these focused on the analysis of *RAS* genes as their alterations are useful to predict the response to anti-EGFR mAb treatment [91,95,96,97,98,99,100,101,102,103]. Spindler et al. were among the first to analyze the concordance of *KRAS* mutation between tissue and cfDNA, obtaining a sensitivity of 78% and absolute specificity, as none of the patients without mutations in primary tumor tissue showed positive plasma samples [95]. Another study reported a concordance of 93% between tissue and plasma analyses in 115 mCRC patients, with the *RAS* mutation status discrepancy explained by spatial and temporal tumor heterogeneity [99]. The authors found that peritoneal and lung metastases, and mucinous histology, were associated with low *RAS* cfDNA detection, whereas the number of metastases was not, suggesting that intrinsic biological characteristics of the tumor rather than tumor burden may impact cfDNA release [99]. Similarly, a concordance of about 90% between tissue and plasma analyses was observed by Grasselli et al. in a case series of 146 mCRC patients. The 10% discordance prevalently referred to cases of *RAS* mutation positivity in cfDNA but negativity in tumor tissue, once more suggesting that such a result could be due to tumor tissue heterogeneity [100]. In another study performed on two independent case series, the positive and negative concordance between plasma and tumor *RAS* mutation was 90.4% and 93.5%, respectively, with an overall concordance of 91.8% [102].

Recently, a very high concordance (97%) was reported in *RAS* mutation between plasma and tumor tissue in mCRC patients with liver metastases. These results confirm the high concordance between primary tissue and liver metastases, suggesting that liquid biopsy could be contemplated for patients in this subgroup for whom no tumor tissue is available [104].

Similarly, a recent study by Normanno et al. on a case series of 92 *KRAS* exon 2 wt patients treated with first-line cetuximab plus FOLFIRI showed a mutation concordance between tissue and cfDNA of 78.3%, with 10 cases of *RAS* mutated in tissue and *RAS* wt in plasma, and a further 10 cases of *RAS* mutated in plasma and *RAS* wt in tissue [103]. Of note, the use of highly sensitive methodologies, such as droplet digital PCR, in discordant cases revealed the presence of mutations in all tissues that were negative by NGS but had matched positive plasma, and in 20% of plasma that was negative by OncoBeam but had matched positive tissue, suggesting that the main reason for the discordance between tissue and cfDNA was the sensitivity of the methodologies, which may not always identify underrepresented alterations. In Normanno’s study, *RAS* mutation in tumor tissue but not in plasma was associated with a better prognosis, implying that low amounts of cfDNA could be indicative of less aggressive disease [103].

### 4.2. Temporal Heterogeneity and Monitoring Response to Therapy

The problem of tumor heterogeneity becomes even more prominent during pharmacological treatment, when a clonal selection develops and a series of secondary resistance mechanisms is established. As tumor re-biopsy is an invasive procedure that may not be feasible, in particular for metastases deep within the body; the only opportunity to investigate and monitor these alterations occurring during treatment is the study of liquid biopsy. 

#### 4.2.1. Anti-EGFR Therapy

**Figure 2:**
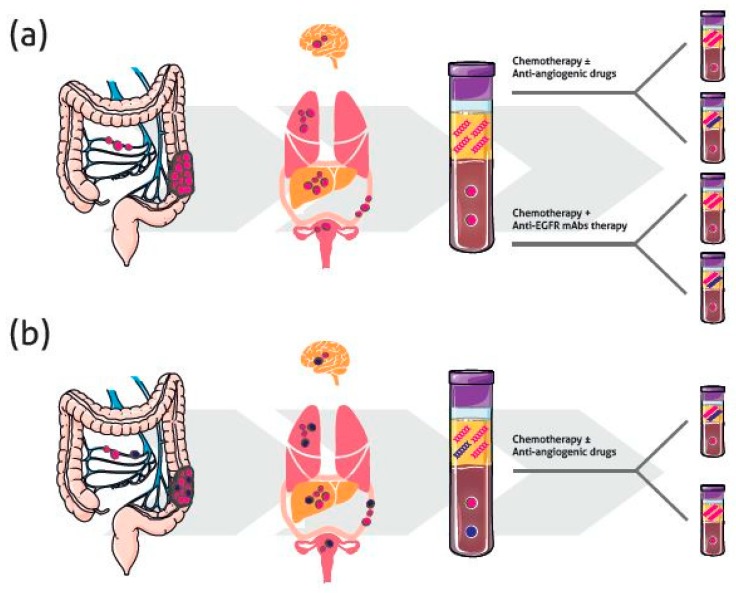
Spatio-temporal heterogeneity and the role of liquid biopsy in the management of mCRC. Example based on *RAS* mutational status analysis. (**a**) *RAS* wild type (wt) tumors. Even in an apparently homogeneous genetic context, the selective pressure exerted by drugs can induce de novo mutations or the selection of mutations present at subclonal level in the primary tumor. (**b**) *RAS* mutated tumors. Heterogeneity can be present within the primary tumor, within metastases, and between primary tumor and metastases. Treatment based on chemotherapy + anti-angiogenic drugs can determine a switch to prevalently wt *RAS* circulating tumor DNA (ctDNA).

In patients treated with targeted therapy, tumor subclones with resistance-conferring aberrations in the target that cannot compete in the absence of therapy will eventually be selected to grow under the treatment pressure. Moreover, de novo mutations may drive resistance to anti-EGFR mAb treatment. Figure 2a summarizes the possible scenario in this patient setting, where *KRAS* mutations represent the principal mechanism of acquired resistance and can be detected non-invasively in blood months before the appearance of radiographic progression [105]. Remarkably, not only *KRAS* alterations, but also *NRAS* and *BRAF* mutations, as well as amplification of *ERBB2* and *MET*, are detected in patients who respond to EGFR blockade and then relapse, highlighting that although the mechanisms of resistance are genetically heterogeneous, they converge on key signaling pathways [106].

In a study by Morelli et al. analysis of plasma collected at progression in 60 patients refractory to anti-EGFR mAbs and *KRAS* wt revealed the presence of *KRAS* mutation in 44% of cases [107]. In 30% of these, mutations were found at a low allele frequency in pre-treatment tumor tissue [107], suggesting that newly detected *RAS* mutation in plasma from patients refractory to anti-EGFR treatment may derive from rare, pre-existing clones in primary tumors. The presence of cfDNA *RAS* mutations was associated with a shorter PFS [107].

In a case series of 20 *RAS-BRAF* wt patients showing progression after anti-EGFR mAbs treatment, NGS analysis revealed that *RAS* mutations and *HER2/MET* amplifications were the most frequently detected resistance mechanisms in both tissue and plasma samples, the authors demonstrating the involvement of both intralesion and interlesion tumor heterogeneity in the emergence of the complex resistance mechanisms [108]. 

In a similar patient setting in which cfDNA was evaluated, Strickler et al. observed multiple tumor subclones characterized by resistance-related alterations emerging during therapy that originate from different metastases throughout the body [109]. 

In another recent study performed on a case series of mCRC patients treated in a phase II trial with panitumumab plus irinotecan, the incidence of *RAS* mutation in tumor tissue was 9.5% by NGS and 6.3% by BEAMing, whereas cfDNA evaluated by BEAMing revealed an incidence of 37%. This result demonstrated that cfDNA was more indicative of resistance mechanisms than tumor tissue, and this is more probably attributable to tumor heterogeneity, which increased dramatically during treatment. Interestingly, *RAS* mutations in cfDNA preceded clinical progression by a median of 3.6 months, indicating that the serial monitoring of *RAS* mutations on cfDNA during treatment could be useful for the timely identification of patients with a high risk of progression [110]. Finally, the highest temporal heterogeneity (94%) was detected by Yamada et al. who reported that 17/18 chemo-naive *KRAS* wt mCRC patients submitted to first-line systemic chemotherapy including EGFR blockade were characterized by the emergence of *KRAS* mutation [111].

Overall, tumor heterogeneity at the time of acquired resistance to anti-EGFR treatment represents a significant obstacle to the development of precision medicine strategies, and suggests the potential usefulness of large-scale cfDNA profiling to define the genomic landscape of tumor heterogeneity and therapeutic resistance.

#### 4.2.2. Chemotherapy and Other Targeted Therapies

Interesting data based on tissue analyses has demonstrated that mutation shifts after chemotherapy may be related to ITH due to tumor clone selection or cancer evolution after chemotherapy. Like targeted therapies, systemic chemotherapies may impose an evolutionary pressure on cancer, thus increasing heterogeneity in terms of new driver and passenger mutations, creating neoantigens that strongly influence immune response [36] (Figure 2a). Conversely, Brannon et al. hypothesized that prior chemotherapy treatment results in a decrease in apparent tumor heterogeneity [69]. A reduction in *RAS/BRAF/PIK3CA* mutations after chemotherapy was observed by Li et al. even when samples with low cellularity, which may give rise to false-negative results, were excluded [52].

Few studies have been published to date on the use of liquid biopsy to evaluate the effect of chemotherapy on heterogeneity. Interestingly, in their preliminary results, Gazzaniga et al. showed that 30% of mutated *KRAS* mCRCs treated with anti-angiogenic drugs switched to prevalently wt *KRAS* ctDNA in peripheral blood during therapy (Figure 2b), probably due to the induction of hypoxia [112]. This is in line with the observation in tissue of Li et al. [52], and if confirmed in larger populations, should be taken into account when a second-line therapeutic option is required. Notably, and in agreement with the above, Yamada et al. recently reported that mutations that emerged in some patients after anti-EGFR mAb treatment disappeared after starting second-line chemotherapy [111]. 

## 5. Impact of Heterogeneity in Clinical Molecular Diagnostics

Considering the importance of molecular targeted therapy in CRC, it is clear that the accurate detection of gene mutations to bypass tumor heterogeneity is essential for correct patient selection. In fact, although it is difficult to predict whether patients with a low frequency of *RAS* mutations will respond or not to anti-EGFR therapy, it is important to identify and characterize all mutant subpopulations during the routine diagnostic process, because their impact on the response to target therapy is still unclear [49]. International guidelines indicate that *RAS* mutation analysis should be performed at diagnosis by analyzing the most recent and representative tumor tissue available [3]. The choice of the specimen and the selection of the appropriate tissue block represent the first challenge, because poor tumor cellularity, ITH, and adjuvant therapy may be confounding factors [52]. Nelson et al. recommended pooling three tumor sites with adequate cellularity for genetic testing to minimize the risk of false negative results [113]. Moreover, since for a subset of mCRC patients the diagnosis of metastases is done only by radiological studies, often only a primary tumor sample is available to perform molecular determinations, even if the principal goal of anti-EGFR therapy is to treat metastases. Despite the fact that the high concordance between primary tissue and metastasis has led to the approval of guidelines indicating that either type of material can be used indifferently, a proportion of patients for whom there is no concordance between primary and metastatic lesions are destined not to receive optimal treatment [14,114]. Moreover, this may be a pitfall for patients with locally advanced CRC who relapse after surgery and adjuvant treatment, as the mutational status of the remaining tumor cells may be altered due to chemotherapy [52]. Similarly, in non-chemo-naive patients, new biopsies of primary or metastatic lesions are probably needed following chemotherapy resistance or insensitivity to fully understand the complexity of the disease and to choose the most appropriate therapy [13]. Overall, when looking at advanced stages of CRC, patients need to be individually oriented due to case-specific prior surgical and clinical treatment and molecular progression. 

## 6. Conclusions

In summary, spatial heterogeneity and drug-selected clonal evolution in CRC influences tumor aggressiveness, resistance to therapy, and patient prognosis. Although a high concordance of the mutational status exists between primary tumor and liver metastases, it appears that the localization of secondary lesions predict the risk of molecular discordance from the primary tumor. Thus, although the use of liquid biopsy in CRC clinical practice is not as urgent as in other malignancies, such as lung cancer, it could represent a tool for further improving the molecular diagnostics of this disease. Moreover, the increasing development of high-throughput sequencing methodologies in both tumor tissue and liquid biopsy, and the gradual decrease in their costs, will provide the possibility of performing genome wide tumor characterization in a non-invasive manner and at any time during treatment, bypassing the problem of tumor heterogeneity and substantially improving mCRC patient management. 

## Figures and Tables

**Table 1:** Concordance between tumor tissue and cell-free DNA (cfDNA) for *RAS*, and *BRAF* mutations in metastatic CRC (mCRC).

Reference	No. Patients	Methodology	Gene	Mutations in cfDNA/Tumor Tissue	Sensitivity/Specificity	Concordance
Spindler et al. [95]	98	qPCR	*KRAS*	34%/43%	78%/100%	NA
Taly et al. [98]	50	ddPCR	*KRAS*	28%/38%	74%/89%	NA
Thierry et al. [101]	95	Intplex qPCR	*KRAS* *BRAF*	39%/38% 6%/6%	92%/98% 100%/100%	96% 100%
Spindler et al. [96]	140	qPCR	*KRAS*	23%/34%	NA	NA
Vidal et al. [99]	115	Beaming	*RAS*	47.8%/51.3%	96.4%/90%	93%
Bachet et al. [104]	406	NGS	*RAS*	42%/55%	92%/94%	93%
Thierry et al. [97]	34 97	Intplex qPCR	*RAS* *BRAF*	12%/9% 14%/7%	67%/94% 57%/89%	92% 87%
Grasselli et al. [100]	146	Beaming	*RAS*	39%/46%	85%/91%	90%
Schmiegel et al. [102]	98	Beaming	*RAS*	51%/53%	90%/94%	91.8%
Normanno et al. [103]	92	NGS	*RAS*	36%/36%	70%/83%	78%

NA: Not available.
